# Plant-based protein extrusion optimization: Comparison between machine learning and conventional experimental design

**DOI:** 10.1016/j.crfs.2025.101157

**Published:** 2025-07-28

**Authors:** Yingfen Jiang, Noor Irsyad Bin Noor Azlee, Wing Shan Ko, Kaiqi Chen, Bee Gim Lim, Arif Z. Nelson

**Affiliations:** a Food, Chemical and Biotechnology Cluster, Singapore Institute of Technology, 1 Punggol Coast Road, Singapore, 828608, Singapore; b Department of Computer Science, National University of Singapore, 13 Computing Drive, Singapore, 117417, Singapore

**Keywords:** Machine learning, Bayesian optimization, Twin-screw extrusion, Plant-based proteins, Tensile strength, Response surface methodology

## Abstract

High-moisture extrusion (HME) is a promising technique for developing fibrous plant-based meat analogues. In HME, protein-water formulations are passed through a heated twin-screw barrel before solidifying in a cooling die, where complex physicochemical transformations occur, making process optimization challenging. Traditional approaches like Response Surface Methodology (RSM) require extensive trials and rely on predefined polynomial models, limiting predictive power. In contrast, Bayesian Optimization (BO), a machine learning technique, uses probabilistic surrogate models to efficiently explore parameter spaces and optimize black-box functions with fewer experiments. This study compares RSM and BO for optimizing the mechanical properties of twin-screw extruded meat analogues to replicate chicken breast by varying barrel temperature, water content, and cooling die temperature. To facilitate a direct comparison, BO was constrained to explore within the dataset employed by RSM, although this restriction may limit BO's full optimization potential. Tensile strength was identified as a key property that improved model fitting and predictive accuracy for both RSM and BO. Compared to the 15 experimental trials required by the RSM approach, BO converged on an optimal set of parameters using only 11 of the 15 RSM trials without tensile strength. When tensile strength was included, the output of only 10 trials was needed before convergence was observed. Experimental validation showed BO predictions had lower a prediction error (≤24.5 %) compared to RSM (up to 61.0 %). These findings highlight the potential of superior predictive accuracy and efficiency in optimizing complex pilot-scale food processing systems such as HME through BO.

## Highlights

• Bayesian optimization (BO) was applied to high-moisture meat-analogue extrusion.
• BO compared favourably to a conventional Response Surface Methodology (RSM) approach.
• The relationship between extrusion conditions and physical properties was built.
• Extrudate tensile strength characterisation improved model prediction capability.
• BO showed better predictability versus RSM despite incorporating fewer experiments.

## Graphical abstract

**Figure:**
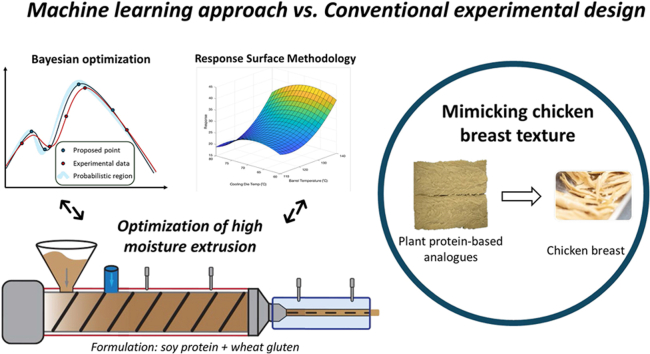

## 1 Introduction

The global food system faces unprecedented challenges in meeting the nutritional demands of a population projected to reach 9.8 billion by 2050 (Nations, 2019). The food sector, particularly animal-based protein production, currently accounts for approximately 30 % of global greenhouse gas emissions (Vermeulen et al., 2012), presenting significant environmental concerns. The environmental impact, combined with increasing protein demands, necessitates a transition toward more sustainable protein sources, with plant-based meat alternatives emerging as a promising solution (Sui et al., 2024).

**Fig. 1:**
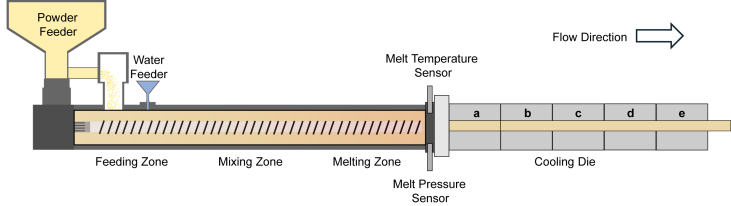
Schematic diagram of a twin-screw extruder with a cooling die. Barrel temperature optimization was performed at zones 8 and 9 (melting zone), while cooling die temperature optimization focused on sections a and b.

While substantial progress has been made in developing ground meat analogues, fabricating structured whole-cut meat alternatives remains a major technological hurdle in the field of alternative proteins (Mateen et al., 2023). The primary challenge lies in replicating muscle tissue's sophisticated hierarchical architecture, which is comprised of aligned protein fibers that create the characteristic textural properties. High-moisture extrusion technology has been extensively utilized to produce fibrous structures from plant proteins, mimicking meat-like textures (J. Zhang et al., 2022). A typical high-moisture twin-screw extruder contains several distinct functional zones—feeding, mixing, melting, and cooling—as illustrated in the simplified schematic in Fig. 1 and detailed representation in Fig. S1. Within the extruder barrel, proteins undergo intensive mixing with water to ensure complete hydration, followed by concurrent denaturation and phase transitions under the combined effects of elevated temperature and high shear forces. As the mixture exits the barrel and enters the cooling die, proteins realign and form structured fibrous textures (Sui et al., 2024).

Optimizing the extrusion process is a significant challenge due to its multifaceted and highly interdependent physicochemical transformations. This complexity is further compounded by the sensitivity of the final product's characteristics to numerous processing parameters. For example, Singh et al. (2024) demonstrated that feed moisture content significantly influenced the textural properties and amino acid profile of sunflower-incorporated meat analogues. Similarly, Pavani et al. (2024) highlighted that barrel temperature played a more substantial role than screw speed in determining the nutritional qualities of pea protein isolate extrudates. Although these studies investigated the individual effects of specific processing parameters, the complexity of the extrusion process is not fully understood. Consequently, extrusion processing is often described as a 'black box' system, where input-output relationships are very difficult to predict.

Response surface methodologies (RSM) have traditionally been used as the primary approach for optimizing extrusion parameters. While proven effective in various applications, including extraction (Pinto et al., 2021), and microencapsulation (Alvarenga Botrel, 2012), RSM has notable limitations. RSM often relies on polynomial models that may oversimplify the non-linear and dynamic nature of the process of interest, and requires extensive experimental trials. In the food industry, particularly when dealing with pilot-scale processes, experiments demand substantial amounts of raw materials, labor, and extensive experimental time. These practical constraints underscore the pressing need for further exploration into advanced and adaptable optimization strategies that can effectively reduce experimental trials while maintaining robust process optimization.

Bayesian optimization (BO) is a machine learning technique that has emerged as a promising alternative to traditional methods (Frazier, 2018). BO leverages probabilistic surrogate models and adaptive sampling strategies to navigate complex parameter spaces efficiently. The surrogate model, typically a Gaussian Process, is continuously refined with each new added data point, and uses an acquisition function to guide the next most informative experimental condition. By adaptively suggesting experimental trials while capturing non-linear dynamics, BO offers a powerful framework for addressing the challenges inherent in extrusion processes. Recent studies have demonstrated this framework's effectiveness in optimizing 3D printing processes, including printing parameters (Etefagh and Razfar, 2024), bioink formulations (Ding et al., 2024) and cell viability post-printing (Rane et al., 2024). For instance, Etefagh and Razfar (2024) demonstrated BO's efficiency in optimizing 3D bioprinting parameters (pressure, speed, temperature) for nanocomposite scaffolds, achieving 91 % prediction accuracy for filament widths while requiring only 11 experimental iterations. Their work also showed BO's capability to handle dynamic parameter adjustments for fabricating gradient structures. Despite these successes in other fields, BO remains largely unexplored for food manufacturing processes, presenting a chance for innovation. A very recent work was published by Theng et al. (2025), demonstrating the effectiveness of BO in high-moisture extrusion optimization. They successfully modeled the relationship between key extrusion parameters—moisture content, barrel temperature, and screw speed—and textural properties including hardness, chewiness, springiness, and cutting force. Their study highlighted the use of BO to fine-tune extrusion conditions to produce plant-based meat analogues with improved texture to mimic that of air-fried chicken breast.

While the recent findings reinforce the promise of applying BO to plant-based meat analogues, there remains an opportunity to further explore its advantages in comparison with traditional optimization methods. In this study, we directly compare BO with RSM to evaluate their effectiveness in optimizing extrusion parameters for a blend of soy-protein concentrate (SPC) and wheat gluten (WG) (X. Zhang et al., 2022). SPC is commonly utilized in high-moisture extrusion due to its balanced amino acid profile and ease of texturization (Li et al., 2005). WG, composed of gliadin and glutenin proteins, complements SPC by forming a cohesive viscoelastic network that enhances structural integrity and fiber stability in meat analogues (Chiang et al., 2019). Our intent here is to reduce the number of required experimental data points to predict the settings which minimize the difference in textural properties between the extrudate and a target food sample. We emphasize that the primary aim of this study is not to precisely replicate the target food sample, but to evaluate the efficiency of BO and RSM in optimizing process parameters toward improved textural properties. While it may limit BO's full predictive potential, in order to facilitate a more direct comparison, the data provided to the BO framework was restricted to the dataset generated by RSM. Conventionally, BO iteratively updates the Gaussian process model with each experimental result and proposes the next most promising condition. However, confining the search to the RSM-defined space restricted the algorithm's ability to explore the broader parameter space and fully harness its exploratory potential. Despite this limitation in the parameter search space, we find that BO still outperforms RSM in terms of model predictive capability and accuracy, even when trained on only a subset of the RSM-generated dataset. In addition to characterizing conventional textural parameters via compression and cutting, we incorporate tensile testing to provide deeper insights into the anisotropic mechanical behavior and fibrous network alignment of plant-based proteins. Our findings also indicate that incorporating tensile properties improves model accuracy and provides a more robust optimization framework. By broadening optimization approaches and evaluation metrics, this study enhances process development for plant-based meat analogues.

## 2 Materials and methods

### 2.1 Raw materials

Soy protein concentrate (SPC; Solcon S100: Protein content: 69.30 %) was purchased from Solbar, Ningbo, China. Vital wheat gluten (WG; Protein content: 74.94 %) was obtained from Roquette, Lestrem, France. Ready-to-eat, herb-flavored chicken breasts (Betagro, Thailand) were purchased from a local convenience store.

### 2.2 High moisture extrusion

Meat analogues were extruded utilizing a pilot scale co-rotating, intermeshing twin-screw extruder, as schematically illustrated in Fig. 1 & Fig. S1 (ZSE 27 MAXX, Leistritz Extrusionstechnik GmbH, Nuremberg Germany). The extruder specifications included a 28.3 mm screw diameter, 1018.8 mm barrel length, and 36:1 length-diameter ratio. The cooling die measured 530 × 120 × 136 mm (L × W × H). The extruder barrel consisted of nine independently controlled heating zones. The cooling die consisted of five independently controlled cooling zones.

The screw configuration incorporated multiple element types: spacer elements (ZD) with 17.1 mm internal diameter in 5 mm or 10 mm widths; various conveying elements including non-self-wiping (GFF), intermeshing (GFA), and mixing (GFM) types; and kneading blocks (KB), with screw element nomenclature formatting given in the extruder instructional manual. The nomenclature for conveying elements specified lobe count, pitch (mm), length (mm), and direction (Re for conveying, L for re-conveying). For example, GFA 2-30-15-L denoted a two-lobed, 30 mm pitch, 15 mm length re-conveying element. KB elements were characterized by disc count, lobe count, length (mm), and offset angle.

The following screw configuration, described using the nomenclature explained above, was applied from feed to discharge and consisted of spacer, conveying, and kneading elements in the following arrangement: 1 × ZD-17.1-10, 1 × ZD-17.1-5, 1 × GFF 2–40-30, 1 × GFF 2–40-30, 7 × GFA 2–40-30, 1 × GFM 2–20-30, 3 × KB 4–2–15-30°, 1 × GFA 2–20-30, 1 × GFA 2–30-30, 3 × KB 4–2–15-60°, 1 × GFA 2–40-30, 2 × GFA 2–30-15, 1 × GFA 2–30-30, 1 × GFA 2–20-30, 1 × GFM 2–20-30, 2 × KB 4–2–15-30°, 3 × KB 4–2–15-60°, 1 × KB 4–2–15-90°, 1 × GFA 2–30-30, 1 × GFA 2–20-30, 1 × GFA 2–30-15-L, 2 × GFA 2–40-30, 2 × GFA 2–30-30 and 3 × GFA 2–20-30.

The extrusion process was conducted at a constant total throughput of 10 kg/h and screw speed of 200 rpm, utilizing a binary mixture of SPC and WG at a mass ratio of 60:40. Preliminary investigations identified process parameters that significantly influenced extrudate quality and established operational boundaries within which stable extrusion could be maintained. Subsequently, three key parameters were prioritized for optimization: barrel temperature (zones 8 and 9, the last two barrel zones), water content, and cooling die temperature (sections a and b, the first two cooling zones). Other parameters such as screw speed and raw material feeding rate were considered for inclusion, but screw speed is reported to be of lesser significance for texturization (Mateen et al., 2023) and preliminary trials found that varying raw material feeding speed within our operational range had a minimal impact on texture. Since the inclusion of these parameters would greatly expand the number of experimental permutations, we leave a more detailed consideration of their impact to future studies.

### 2.3 Experimental design

A Box-Behnken design (BBD) was selected for producing the RSM dataset because it avoids extreme factor-level combinations, reducing the risk of impractical or unstable extrusion conditions while ensuring that experiments remain within the feasible operational space. A three-factor, three-level design was implemented using MATLAB (R2024a, The MathWorks, Natick, MA, United States) to investigate the influence of processing variables on extrudate characteristics. The independent variables were barrel temperature (110–140 °C), water content (55–65 %), and cooling die temperature (60–80 °C), as detailed in Table 1. The first 7 sections of the heated barrel were maintained at consistent temperatures across all runs (35, 50, 70, 90, 115, 125, and 125 °C), while the temperatures in sections 8 and 9 varied according to Table 1. Similarly, the cooling die section temperatures were maintained at temperatures of 50, 50 and 40 °C in sections c-e, respectively, with variable temperatures in section a and b as shown in Table 1. Following the BBD, the experimental matrix consisted of 15 runs, including 3 replications of the center points, with variables coded at three levels (−1, 0, +1).

**Table 1:** Experimental design layout using Box Behnken Design.

Run	Coded values			Actual values		
	Barrel Temperature	Water Content	Cooling Die Temperature	Barrel Temperature (°C)	Water Content (%)	Cooling Die Temperature (Section a, b) (°C)
1	−1	−1	0	110	55	70, 60
2	1	−1	0	140	55	70, 60
3	−1	1	0	110	65	70, 60
4	1	1	0	140	65	70, 60
5	−1	0	−1	110	60	60, 55
6	1	0	−1	140	60	60, 55
7	−1	0	1	110	60	80, 65
8	1	0	1	140	60	80, 65
9	0	−1	−1	125	55	60, 55
10	0	1	−1	125	65	60, 55
11	0	−1	1	125	55	80, 65
12	0	1	1	125	65	80, 65
13	0	0	0	125	60	70, 60
14	0	0	0	125	60	70, 60
15	0	0	0	125	60	70, 60

Samples were collected after achieving steady-state operation, defined by concurrent stability criteria: melt temperature variations within ±2 °C, melt pressure fluctuations within ±10 %, and motor torque oscillations within ±2 %, maintained for a minimum duration of 5 min. The extruder system conditions during sample collection are detailed in Table S1. The collected extrudates were immediately sealed in cling wrap to prevent moisture loss and stored at −20 °C. Before physical characterization, extrudates were defrosted overnight at 4 °C and then equilibrated at room temperature for approximately 1 h while remaining wrapped.

### 2.4 Physical properties of extrudates

#### 2.4.1 Sample preparation

**Fig. 2:**
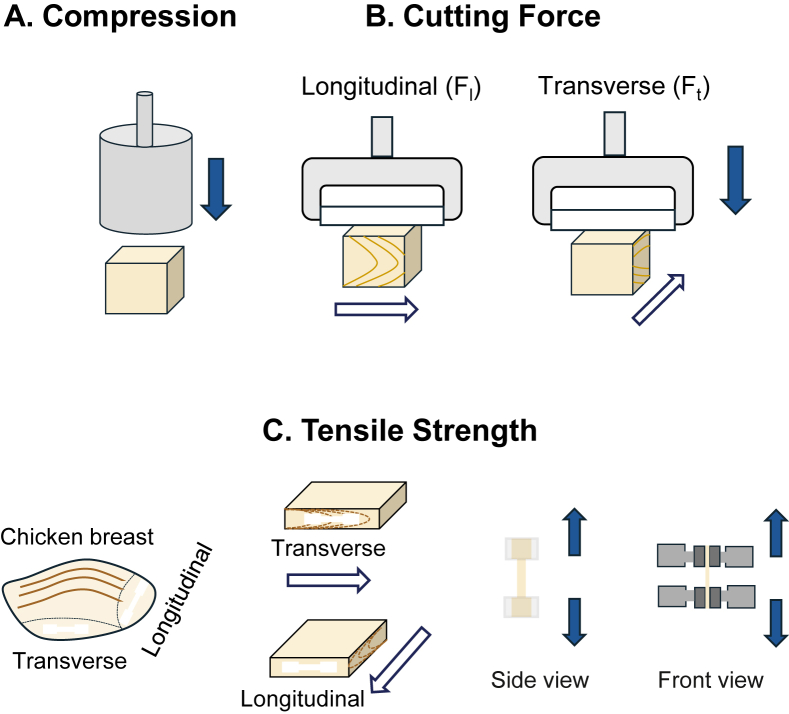
Sample preparation for physical evaluations of A. compression, B. cutting force and C. tensile strength.

For compression and cutting force tests, extruded plant-based meat analogues were cut into cubes with an edge length of 12 mm. For the tensile strength test, specimens were prepared from the meat analogues in both longitudinal and transverse orientations relative to the extrusion flow direction. A dog-bone geometry, was employed to localize stress within the central region of the specimen (Taghian Dinani, 2023). The dimensions of the geometry are detailed in Fig. S2, while the schematics of the sample preparation process are shown in Fig. 2A–C.

#### 2.4.2 Compression test

The hardness, springiness, and chewiness of plant-based meat analogues and the chicken breast were double compressed by a texture analyzer (TA.XT Plus C; Stable Micro Systems, Godalming, England) with a P/36R probe based on an adaptation of the method of Jiang et al. (2024). The test was performed in triplicate at a 5 mm/s testing speed and 60 % strain.

#### 2.4.3 Cutting force determination

The cutting force strength was measured using a texture analyzer (TA.XT Plus C; Stable Micro Systems, Godalming, England) equipped with an extended craft knife blade (A/ECB) probe, adapting the method described by Tan et al. (2024). The maximum force required to cut the samples to 90 % of their thickness along the transverse (F_T_) or longitudinal (F_L_) direction relative to the extrusion flow direction was recorded as the cutting force. This value was used as an indicator of cutting toughness. All experiments were conducted in triplicate.

#### 2.4.4 Tensile strength analysis

Tensile strength was measured using a texture analyzer (TA.XT Plus C; Stable Micro Systems, Godalming, England) equipped with an A/MTP mini tensile grip. This method was adapted from Schlangen et al, (2023). Samples were clamped securely in the grips and stretched at a constant test speed of 1 mm/s until complete failure occurred. The maximum force required to break the sample was recorded as the tensile strength. The maximum tensile strength of samples was measure in the transverse (T_T_) and the longitudinal (T_L_) orientations relative to the extrusion flow direction. The test was performed in triplicate.

### 2.5 Modelling

#### 2.5.1 Multi-objective optimization using weighted-sum method

To transform the multi-dimensional optimization problem into a tractable single-objective formulation, a weighted-sum methodology was implemented. Due to the absence of strong empirical evidence supporting differential weighting of the textural parameters, equal weights were assigned to each variable during the optimization process. The objective function was constructed as an aggregated measure of normalized deviations from a reference point, utilizing ready-to-eat chicken breast as the target. This approach optimizes multiple quality parameters while reducing computational complexity. The scalarization function is defined as,

(1) $$S\left(\theta \right)=\sum_{i=1}^{n}\frac{\left|x_{i}\left(\theta \right)-x_{i,ref}\right|}{x_{i,ref}}$$

Where *S*(θ) denotes the scalarized value to be used for the modeling, θ denotes the process parameters that may affect the output quality, $x_{i}$ denotes the measured value of the *i*^*th*^ variable, which is a function of θ, $x_{i,ref}$ denotes the reference value for the *i*^*th*^ variable based on the chicken breast standard, and *n* is the total number of variables included in the scalarization.

This formulation quantifies the cumulative normalized deviation from the reference state across all parameters. The optimization problem is to minimize *S*(θ), i.e. to identify the process parameters which produce an output which has the smallest scalarized difference with the target.

#### 2.5.2 Response surface methodology

To provide a reference solution to the above optimization problem, a widely applied method called Response surface methodology (RSM) was employed. In this method, the relationship between the independent variables and responses was modeled using a second-order polynomial equation as shown in Eq. (2):

(2) $$\hat{S}=\beta_{0}+\sum_{i=1}^{n}\beta_{i}\theta_{i}+\sum_{i=1}^{n}\beta_{ii}\theta_{i}^{2}+\sum_{i=1}^{n-1}\sum_{j=i+1}^{n}\beta_{ij}\theta_{i}\theta_{j}$$

where $\hat{S}$ represents the predicted response; *β*_*0*_ is a bias term; *βᵢ*, *βᵢᵢ*, and *βᵢⱼ* denote the coefficients for linear, quadratic, and interaction terms, respectively; and $\theta_{i}$ and $\theta_{j}$ are i th and j th elements of process parameter array θ.

Statistical validation of the models was performed using ANOVA, and model fitness was evaluated through the adjusted coefficient of determination (*R*^*2*^_*adj*_) and lack-of-fit testing. Contour plots were generated to visualize the interactive effects of process variables on the responses and to identify optimal processing conditions.

As captured in Equation (2), RSM assumes that the process response is approximately quadratic. However, real-world food processing responses are often complex and non-linear. Moreover, RSM requires a specific design for data collection (Durakovic, 2017), which significantly increases the number of experiments required to achieve a working model results.

#### 2.5.3 Surrogate-assisted Bayesian optimization

Bayesian Optimization (BO) has been proposed as a remedy to processes which are not suited for Design of Experiment approaches (Sano et al., 2020), such as RSM, which rely heavily on predefined experimental designs. By using a probabilistic surrogate model to predict process outcomes and guide further experimentation, BO addresses the problem of finding the optimum of an unknown process response function that is typically expensive to evaluate. In this study, BO iterates through 2 steps: 1) Given new experimental data at a given set of process parameters, together with the previously collected data, a Gaussian Process (GP) model is constructed. 2) Based on this GP model, a prediction of the optimal process parameters is made, and to further improve the model the next process parameter to explore is determined (e.g., by comparing expected improvement of different parameters).

i. Step 1: Fitting the Gaussian Process surrogate model

The BO framework employs a GP as its surrogate model with a composite kernel which combines a Constant Kernel (C) and a Radial Basis Function (RBF) kernel. For any process parameter *θ*, the GP provides both a predicted mean *μ(θ)* and uncertainty estimate *σ(θ)*: $S\left(\theta \right)\;∼\;GP\left(\mu \left(\theta \right),k\left(\theta ,\theta^{\prime }\right)\right)$, where $k\left(\theta ,\theta^{\prime }\right)$ is the covariance function (kernel) that defines the similarity between points. The kernel configuration is defined as:

(3) $$k\left(\theta ,\theta^{\prime }\right)={C\cdot \text{RBF}=\sigma }^{2}\;\exp\left(-\frac{{\left|\left|\theta -\theta^{\prime }\right|\right|}^{2}}{{2l}^{2}}\right)$$

Where $\sigma^{2}$ is the signal variance and *l* is the length scale for each parameter dimension.

ii. Step 2: Sequential point selection

Given our objective of minimizing the distance S(θ) (Eq. (1)) between the output and target, a modified Expected Improvement (EI) acquisition function was employed. For clarity, we use $\overset{ˇ}{S}\left(\theta \right)$ to represent the predicted objective function from GP and use S(θ) for the ground truth objective function.

The next experimental point is selected by minimizing the EI:

(4) $$EI\left(\theta \right)=E\left[\min\left(\overset{ˇ}{S}\left(\theta \right)-\overset{ˇ}{S}\left(\theta_{best}\right),0\right)\right]$$

Where $\theta_{best}$ is the optimal process parameters under the predicted objective function from the previous iteration step. The EI can be computed analytically as:

(5) $$EI\left(\theta \right)=\left(\mu \left(\theta \right)-\overset{ˇ}{S}\left(\theta_{best}\right)\right)\Phi \left(Z\right)+\sigma \left(\theta \right)\phi \left(Z\right)$$

Where $Z=\frac{\left(\mu \left(\theta \right)-\overset{ˇ}{S}\left(\theta_{best}\right)\right)}{\sigma \left(\theta \right)}$; Φ and ϕ are the cumulative distribution and probability density functions of standard normal distribution, respectively; μ(θ) is the GP predicted mean error at point θ; σ(θ) is the GP predicted standard deviation (SD) at point θ.

The next process parameter to explore is selected as:

(6) $$\theta^{*}=\mathrm{argmin}\;\left[EI\left(\theta \right)\right]$$

This function inherently balances exploitation of promising regions through the *μ(*θ*)* term with exploration of uncertain areas through the σ(θ) term.

iii. Iterative optimization and validation experiments

**Fig. 3:**
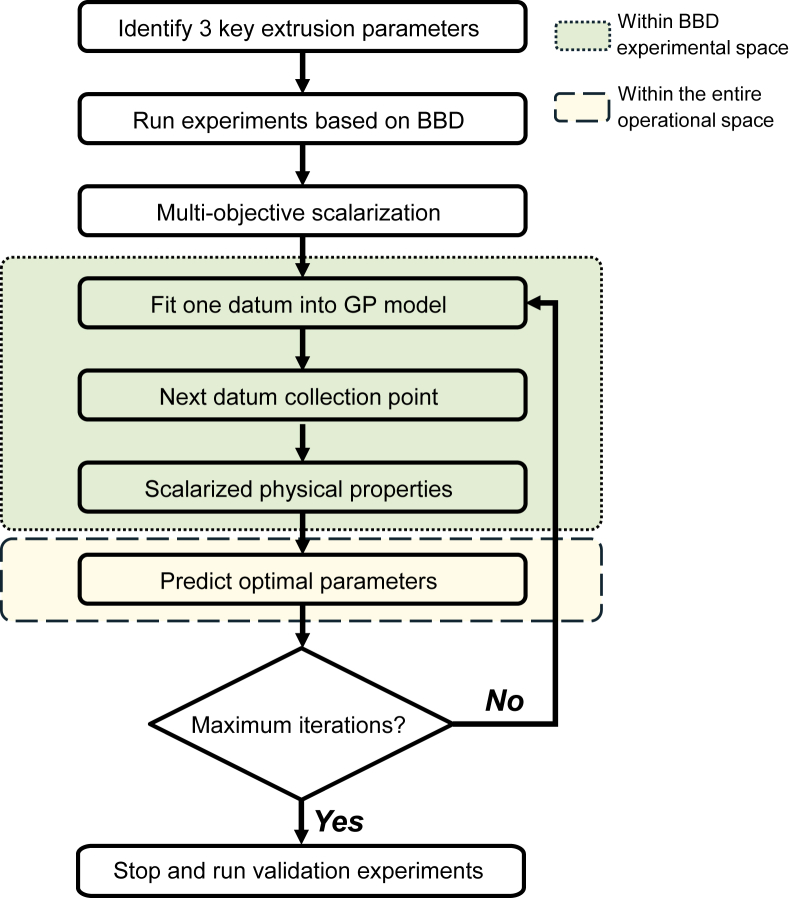
Illustration of the proposed hybrid Bayesian Optimization with response surface methodology.

Fig. 3 illustrates the BO framework for process parameter optimization. The workflow began by identifying three key extrusion process parameters, followed by experiments based on RSM-BBD (Table 1). After multi-objective scalarization of the results, the framework iteratively built predictive capability by fitting experimental data into a GP model. To ensure an unbiased starting point, the center point from the RSM-BBD was selected as the initial data point for BO. Additionally, to account for experimental noise, the variance of the center point replicates was used to set up the BO kernel. Specifically, the alpha parameter, a hyperparameter added to the diagonal of the kernel matrix to model observation noise, was set to the square of the center point's standard deviation. In each subsequent iteration, the algorithm computed EI(θ) across the parameter space and identified the optimal next point $(\theta^{*}$) that minimizes EI(θ). We retrieved the corresponding experimental results *S* from the pre-existing RSM-BBD dataset, and the GP model was then updated with this additional data point. During optimization, the exploration was confined to the RSM-defined parameter space, yet the estimation of optimal parameters extended across the entire operational range. The iterative process continued until the maximum iteration limit was reached, culminating in validation experiments to confirm the accuracy and robustness of the predicted optimal parameters. The Python code used to implement the BO algorithm is available at https://github.com/Renee-Kong/Bayesian-optimization-vs.-RSM.

### 2.6 Statistical analysis

Experimental values are expressed as mean ± SD and analyzed using one-way ANOVA with Tukey's Honest Significant Difference test in R (version 4.4.3, R Core Team, Vienna, Austria). The data points were auto-scaled – each variable was mean-centered and divided by its own standard deviation – to normalize variations between properties with large differences in magnitude before multivariate analysis. Principal component analysis (PCA), partial least squares discriminant analysis (PLS-DA) and Hierarchical clustering heatmap were performed using MetaboAnalyst 6.0. (https://www.metaboanalyst.ca/).

## 3 Results and discussion

### 3.1 Comparison of models incorporating different physical properties

Experimental results of different runs of extrusion experiments under varying conditions are summarized in Table S2. Among three physical evaluation methods, compression and cutting properties are critical indicators for food products and have been widely adopted in extrudate characterization (Tan et al., 2024). They reflect the effectiveness of protein texturization and correlate with sensory attributes such as chewiness and firmness (Mathoniere et al, 2000). To further enhance our understanding of the structure-process relationship in plant protein extrusion, we also expanded our analysis to tensile properties, which have been explored with meat analogues (St. Pierre et al., 2024), but far less often compared to compression and cutting properties. This additional mechanical parameter may provide insights into fibrous structure formation, a key characteristic of meat-like texture (Zink et al., 2024).

#### 3.1.1 Performance of response surface methodology

To assess the significance of extrusion condition variables, ANOVA was performed to fit second-order polynomial RSM models (see Eq. (2)). As shown in Table 2, the first candidate model, which focused on compression and cutting properties only (referred to as “w/o tensile”), was statistically significant (*p* = 0.002, F = 10.53), indicating that the studied factors explained a substantial portion of the response variability. Among the linear effects, water content had the most significant impact (*p* < 0.001, F = 49.80), followed by barrel temperature (*p* = 0.038, F = 6.18). This highlights the dominant role of moisture in plasticizing plant proteins during extrusion, affecting extrudates’ textural firmness (Hu et al., 2024; Z. Zhang et al., 2023). In contrast, cooling die temperature was not significant (*p* = 0.214, F = 1.83), suggesting it has minimal influence on compression and cutting properties within the tested range. This observation contradicts the findings reported by Choi et al. (2025), who demonstrated that increasing the cooling die temperature resulted in reduced extrudate hardness. This discrepancy may be attributed to differences in the raw materials, as well as variations in the applied cooling die temperature range. The lowest cooling die temperature (55 °C) employed in our study is just slightly lower than the highest temperature (60 °C) investigated by Choi et al. (2025), potentially accounting for the inconsistent outcomes. Further analysis revealed that the squared effects of all three factors were statistically insignificant. Given the lack of significance in two-way interaction terms, they were excluded from the final model to prevent overfitting, which occurs when a model captures noise instead of underlying patterns, reducing its generalizability. The goodness-of-fit measures of the adjusted R^2^ of 80.3 % and predictive R^2^ of 61.4 % indicate a less than desirable model fit with poor predictive accuracy.

**Table 2:** Summary of Analysis of Variance (ANOVA) results for response surface methodology.

Source	DF	SS	MS	F-Value	P-Value
Model without tensile strength	6	281.673	46.945	10.53	**0.002**
Linear:	3	257.678	85.893	19.27	**0.001**
Barrel Temperature	1	27.565	27.565	6.18	**0.038**
Water Content	1	221.972	221.972	49.80	**<0.001**
Cooling Die Temperature	1	8.141	8.141	1.83	0.214
					
Square:	3	23.994	7.998	1.79	0.226
Barrel Temperature	1	10.499	10.499	2.36	0.163
Water Content	1	1.003	1.003	0.23	0.648
Cooling Die Temperature	1	10.718	10.718	2.40	0.160
Error	8	35.657	4.457		
Lack-of-Fit	6	24.114	4.019	0.70	0.691
Pure Error	2	11.542	5.771		
Total	14	317.329			
R^2^	88.80 %				
R^2^(adj)	80.30 %				
R^2^(pred)	61.42 %				

Model with tensile strength	6	3587.77	597.96	21.70	**<0.001**
Linear:	3	3221.01	1073.67	38.96	**<0.001**
Barrel Temperature	1	451.35	451.35	16.38	**0.004**
Water Content	1	2730.24	2730.24	99.06	**<0.001**
Cooling Die Temperature	1	39.43	39.43	1.43	0.266
					
Square:	3	366.75	122.25	4.44	**0.041**
Barrel Temperature	1	131.40	131.40	4.77	0.061
Water Content	1	135.84	135.84	4.93	0.057
Cooling Die Temperature	1	84.26	84.26	3.06	0.119
Error	8	220.49	27.56		
Lack-of-Fit	6	151.80	25.30	0.74	0.674
Pure Error	2	68.69	34.35		
Total	14	3808.26			
R^2^	94.20 %				
R^2^(adj)	89.90 %				
R^2^(pred)	80.00 %				

Note: DF: Degree of freedom; SS: Sum of square; MS: Mean square. Bolded *p*-values in the last column indicate statistical significance (*p* < 0.05).

The second candidate model which incorporated tensile properties (referred to as “w/tensile”) was highly significant (*p* < 0.001), reinforcing the strong relationship between extrusion parameters and mechanical properties. Similar to the first model, water content had the strongest influence (*p* < 0.001, F = 99.06), followed by barrel temperature (*p* = 0.004, F = 16.38). Notably, the quadratic effects of water content (*p* = 0.057, F = 4.93) and barrel temperature (*p* = 0.061, F = 4.77) approached significance. This contrasts with the first model, where all quadratic effects were clearly insignificant. Moreover, the adjusted R^2^ (89.9 %) and predictive R^2^ (80.0 %) are substantially higher, indicating that this model has stronger predictive accuracy compared to the compression/cutting model.

**Fig. 4:**
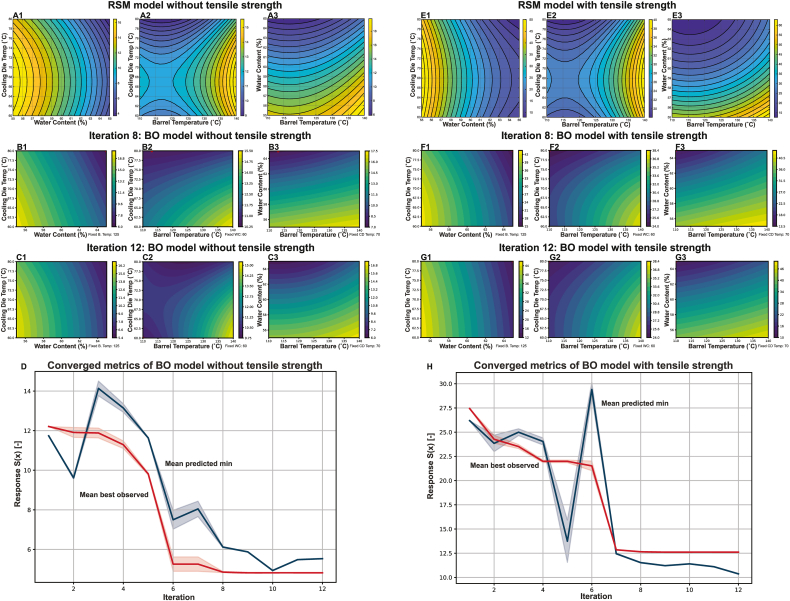
Contour plots (A, B, C, E, F & G) and evolution of the performance metrics with the number of iterations (D & H). Plots A&E are generated from Response Surface Methodology, while B, C, F & G are from Bayesian optimization, with a red arrow showing the predicted optimal condition. Panels A–D represent models excluding tensile properties, whereas E–H include tensile properties. The solid lines in plots D & H are the mean value, and the shaded regions are 95 % confidence interval across 50 runs. B. Temp: Barrel temperature; CD Temp: Cooling die temperature; WC: Water content.

The polynomial equations derived from the RSM analysis for models without and with tensile strength included are presented in Equations (7), (8), respectively, while the contour plots in Fig. 4(A&E) illustrate the combined effects of the key variables on the response. These plots were generated by varying two factors while holding the third at its central value, allowing visualization of how extrusion parameters influence textural outcomes.

(7) $$\hat{S}=25.5694-1.7499BT+1.4485WC+2.2844CDT+0.0075BT^{2}-0.0209{WC}^{2}-0.0170{CDT}^{2}$$

(8) $$\hat{S}=1255.7035-6.1277BT+32.8115WC+6.4678CDT+0.0265BT^{2}-0.2426{WC}^{2}-0.0478{CDT}^{2}$$

Overall, the results indicate that the texture of the meat analogues was primarily influenced by water content, with barrel temperature also playing a significant role. The dominant effect of water content suggested that higher water input may contribute to softer meat analogues, similar to the ready-to-eat chicken breast. These findings align with those of Xia et al. (2023), who reported comparable effects of water content and processing temperature on the texture of yeast protein-based high-moisture meat analogues.

#### 3.1.2 Bayesian optimization results

While the RSM models do provide meaningful insights into extrusion parameter effects, their predictive accuracy remains limited, especially for tensile properties where potential non-linearity was observed. To better capture complex interactions, BO was applied for a more adaptive and data-driven approach to optimizing fibrous texture formation.

Since BO is probabilistic in nature, different runs of the workflow depicted in Fig. 3 may result in different convergence behavior. Fig. 4D and H show the convergence metrics of BO over 12 iterations, comparing the optimization process for models (D) without tensile strength and (H) with tensile strength. Each graph shows the mean predicted minimum curve (blue) and the mean best experimental response (red) with shaded regions indicating the 95 % confidence intervals (95 % CI); these curves were derived from 50 independent BO runs to ensure the robustness of the probabilistic method. Both models exhibited a clear downward trend, confirming BO was effective in identifying improved extrusion conditions. In addition, the narrowing of the 95 % CI in both models towards the later iterations reflected BO's ability to reduce uncertainty as the search space was progressively refined. However, key differences emerged in the optimization trajectory and the number of iterations required for convergence. The w/o tensile model (D) showed a more stable and gradual decline, reaching a steady state at iteration 8. In contrast, the w/tensile model (H) displayed greater fluctuations, with a notable spike at iteration 6 before stabilizing. This oscillation likely reflects a more complex and rugged optimization landscape, where tensile properties introduced additional variability due to their dependence on fiber formation, alignment, and connectivity rather than just bulk gelation mechanisms (Nisov et al., 2022). Despite these fluctuations, the w/tensile model (Fig. 4H) converged slightly earlier (iteration 7) with 10 experimental trials, while the w/o tensile model (Fig. 4D) required 11 trials, converging at iteration 8. The subsequent iterations served to further refine the model until all RSM data points had been incorporated.

The contour plots generated from BO are presented in Fig. 4, comparing model scenarios that do not incorporate tensile strength data (w/o tensile; plots B & C) and model scenarios which do incorporate tensile strength data (w/tensile; plots F & G). Plots B and F represent the initial convergence points, while plots C and G depict the responses at final iteration. Overall, the contour plots displayed qualitatively similar patterns, except for the relationships between barrel temperature and cooling die temperature at fixed water content.

### 3.2 Model robustness and optimization efficiency

To evaluate model robustness, the experimental values from the BBD dataset (using response data including tensile testing) were compared to the predicted values from the RSM and BO models as shown in Table 3. From Table 3, some variability can be observed from the dataset. Although both models demonstrated reasonable predictive capabilities, notable discrepancies were observed in certain trials. For example, the center point replicate runs (13, 14, and 15) which were performed at random intervals throughout the extrusion trials period to assess experimental variability, showed differences, particularly run 15, which varied more notably from the other two replicates. Moreover, run 2 which was conducted under extreme processing conditions, yielded the highest response value of 73.10, while the rest of the dataset responses varied from 12.61 to 47.54.

**Table 3:** Experimental and predicted scalarized values of extrudates from RSM and BO models.

Run	Barrel Temperature (°C)	Water Content (%)	Cooling Die Temperature (Section a, b) (°C)	Experimental (−)	Predicted (−)	
					RSM	BO
1	110	55	70, 60	47.54	50.43	40.51
2	140	55	70, 60	73.10	65.45	48.14
3	110	65	70, 60	12.86	13.48	11.62
4	140	65	70, 60	24.37	28.51	21.38
5	110	60	60, 55	23.85	23.33	26.62
6	140	60	60, 55	39.69	38.36	37.40
7	110	60	80, 65	21.89	18.89	24.44
8	140	60	80, 65	29.07	33.91	30.98
9	125	55	60, 55	47.43	49.42	45.17
10	125	65	60, 55	12.61	12.47	16.71
11	125	55	80, 65	42.21	44.98	40.57
12	125	65	80, 65	12.65	8.03	12.64
13	125	60	70, 60	31.90	27.44	29.40
14	125	60	70, 60	29.61	27.44	29.40
15	125	60	70, 60	20.80	27.44	29.40

RSM and BO models differ significantly in their handling of experimental noise. RSM uses a fixed, least-squares regression approach that assumes homoscedastic (constant variance), normally distributed noise across the design space. The noise is implicitly incorporated into the regression model which is fit by minimizing the least squares criterion. However, polynomial regression in RSM has been shown to struggle with capturing complex interactions since it assumes a predefined functional form which limits adaptability to nonlinear relationships (Kang et al., 2025). Noise estimated from repeated data points reduces the signal-to-noise ratio, therefore lowering the predictive R^2^ and widening the prediction intervals. In contrast, in the BO framework, the GP model handles noise using a likelihood function that optimizes the probability of observing the data given the noise parameters. In this study, the alpha parameter, which represents the noise level in the Gaussian Process kernel (Liu et al., 2020), was set to be equal to the variance of the center point replicates, reflecting the experimental measurement uncertainty. This parameter controls the strength of the regularization, which stabilizes the covariance matrix by adding noise variance to its diagonal. This sacrifices model fit to the noisy training data for better generalization to unseen data, preventing overfitting. In this application, the experimental noise level was explicitly programmed into the BO algorithm as it would be reasonable to assume homoscedasticity in the data. However, in future work, alternative kernels such as a WhiteKernel, which learns the noise level during training, could be employed if larger datasets are available or heteroscedasticity is suspected (Audus et al., 2022). The trade-off of avoiding overfitting can be seen in Table 3 where the predictions for the parameters of run 2, a highly variable trial, widely differ between the RSM and BO models. The larger error from the BO model demonstrates the effect of the regularization in the GP regression, prioritizing global generalization over local accuracy.

The optimal extrusion process parameters obtained from the different models, as well as the predicted and experimental values, are summarized in Table S3 and Table 4, respectively. While the target water content and cooling die temperature remained consistent across all predicted conditions from three models under two different output scenarios, differences were observed in the barrel temperature. Among the set of optimal barrel temperatures recommended, BO7/12 from w/tensile models produced identical predictions and the lowest value of 110 °C. RSM and BO12 from the w/o tensile model predicted optimal barrel temperatures of around 115 and 121, respectively. The comparison of the prediction errors between the RSM and BO models in Table 4 shows that the BO model made significantly more accurate predictions for the optimal parameters. Prediction errors from the RSM model ranged from 44.14 % to 61.00 %, whereas errors from the initial converged BO model ranged from 7.59 % to 14.19 %, and those from the fully iterated BO models ranged from 10.69 % to 24.48 %. The slightly higher errors of the fully iterated BO models may be due to the exploration of sparsely sampled regions in the design space. While this may lead to a temporary increase in error, it is expected to enhance model generalizability and robustness as more data points are incorporated. The comparable performance between the initial converged and fully iterated BO models further highlighted BO's potential to achieve strong predictive accuracy while significantly reducing the required number of experimental trials compared to RSM.

**Table 4:** Predicted and experimental output values under predicted optimal conditions.

Trial	Barrel Temperature (°C)	Water Content (%)	Cooling Die Temperature (Section a, b) (°C)	Experimental (−)	Predicted (−)					
					RSM		BO7/8		BO12	
					Prediction	Prediction error (%)	Prediction	Prediction error (%)	Prediction	Prediction error (%)
Modeling without tensile strength										
RSM	117	65	80, 65	7.12	3.20 ± 1.47	55.06	6.11 ± 1.74	14.19	5.79 ± 1.72	18.70
BO8	113	65	80, 65	6.66	3.30 ± 1.51	50.45	6.08 ± 1.88	8.71		
BO12	121	65	80, 65	7.46	3.33 ± 1.48	55.36			5.69 ± 1.66	23.73
Modeling with tensile strength										
RSM	115	65	80, 65	14.54	5.67 ± 3.70	61.00	13.10 ± 5.93	9.90	10.98 ± 4.18	24.48
BO7	110	65	80, 65	11.60	6.48 ± 4.22	44.14	12.48 ± 5.99	7.59		
BO12	110	65	80, 65	11.60	6.48 ± 4.22	44.14			10.36 ± 4.86	10.69

Note: RSM: response surface methodology; BO: Bayesian optimization. BO7/8/12: The 7^th^/8^th^/12^th^ iteration from Bayesian optimization.

To caveat and contextualize our results, there are potential pitfalls in the application of machine learning techniques compared to RSM. Studies by Solih et al. (2025) and Kang et al. (2025) compare polynomial regression in RSM with machine learning approaches such as Random Forest (RF). Both studies generally agree that machine learning models can offer better predictive performance compared to RSM. However, they also raise the concern that the flexibility of machine learning models, though advantageous for complex datasets, makes them more prone to overfitting. Kang et al. (2025) discussed the risk of overfitting especially when limited data was available or parameters, such as decision tree depth for RF, were not appropriately tuned. Therefore, although the literature indicates that machine learning approaches like BO can outperform RSM in predictive accuracy, these methods also have the potential risk of performing worse than RSM if the important precautions such as parameter tuning and validation are not properly considered.

### 3.3 Process-structure-property relationships

**Fig. 5:**
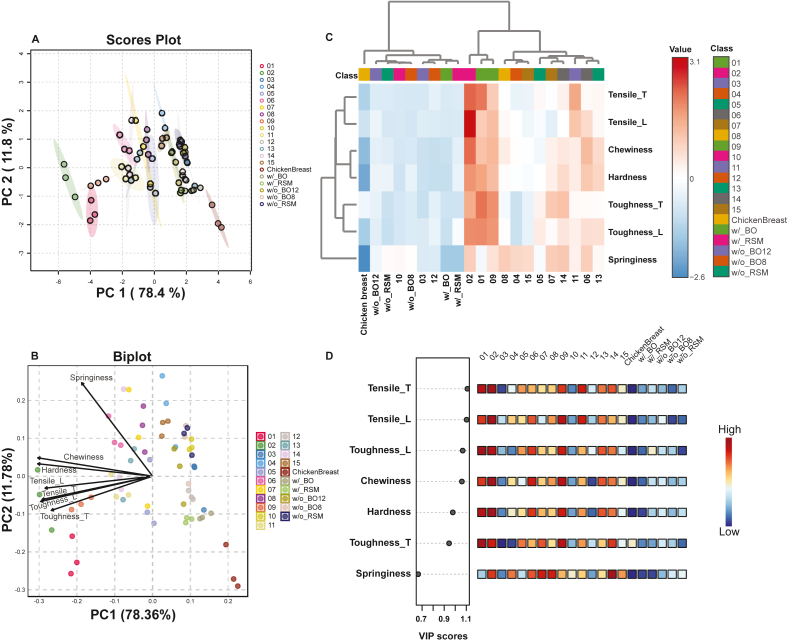
Principal Component Analysis (PCA; A–B) and Partial Least Squares Discriminant Analysis (PLS-DA; C) were performed across all extrudate groups, along with a hierarchical clustering heatmap (D) illustrating variations across all extrudate groups.

**Fig. 6:**
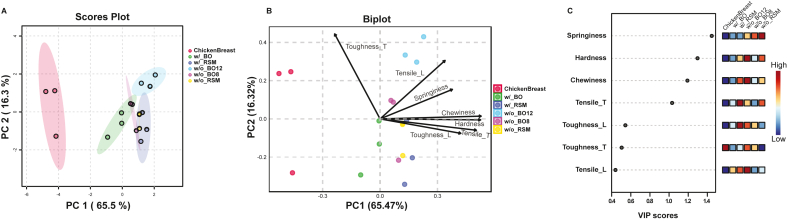
Principal Component Analysis (PCA; A–B) and Partial Least Squares Discriminant Analysis (PLS-DA; C) were performed across optimized groups.

The multivariate statistical analysis shown in Fig. 5, Fig. 6 depicts the complex relationships between processing conditions and the resulting structural and textural properties of the investigated plant-based protein extrudates using principal component analysis (PCA), partial least squares discriminant analysis (PLS-DA) and hierarchical clustering heatmap.

Through PCA, it was found that principal component 1(PC1) accounted for a substantial 78.4 % of the total variability, and PC2 explained 11.8 %, capturing the majority of the dataset's variability between these two components. The PCA scatter matrix of the first five principal components of the full dataset is given in Supplemental Information, Fig. S3A. Fig. 5A provides a more focused visualization of sample distribution along the first two components. The colored ellipses represent 95 % confidence regions, illustrating the clustering of samples under different extrusion conditions. Notably, the chicken breast reference was distinctly separated from the extrudates, positioned on the right side of the PC1 axis. Fig. 5B displays the biplot, overlaying the scores plot with vectors representing PCA loadings for the measured textural properties. The positioning of samples along these principal components revealed distinct textural trends. Springiness strongly differentiates samples along PC2, whereas chewiness, hardness, tensile properties, and toughness predominantly influence variation along PC1. The direction of each arrow indicates the correlation between the textural properties and principal components; properties oriented closely together (e.g., chewiness and hardness) are positively correlated, while those oriented orthogonally indicate no correlation, such as springiness and toughness_T.

While PCA captures the overall variation in the dataset without considering class labels, PLS-DA, as a supervised technique, focuses on maximizing group separation, providing additional insights into key attributes that differentiate extrusion conditions (Uarrota et al., 2014). The left panel of Fig. 5C shows the VIP scores of each parameter, quantifying their relative importance in sample discrimination, with a score above 1.0 considered significant. Tensile properties in both directions ranked highest in VIP scores, aligning with our findings that tensile strength notably enhanced model fitting and predictive performance. In contrast, springiness exhibited the lowest VIP score, indicating its limited discriminative capability. This observation is consistent with results from one-way ANOVA (Table S2), which revealed fewer significant differences in springiness among samples. The associated heatmap (Fig. 5C) provides visual clarity regarding the relative intensity of each texture property across different samples. Chicken breast samples consistently showed distinct textural profiles compared to plant-based analogues, particularly in parameters with high VIP scores. Optimized samples displayed texture profiles more similar to chicken breast than non-optimized samples, underscoring the effectiveness of optimization approaches in achieving targeted textures.

The heatmap shown in Fig. 5D visualizes the clustering of samples based on Euclidean distances of textural properties. Chicken breast samples formed a distinct cluster, characterized by lower values for textural parameters compared to plant-based analogues. Optimized samples (BO and RSM) grouped closer to chicken breast, aligning with expectations. Tensile strength and toughness properties drove the primary cluster separations, highlighting their importance in differentiating sample groups. Conversely, springiness exhibited less variation, indicating lower discriminative significance, consistent with earlier PLS-DA findings (Fig. 5C).

To compare the predictive effectiveness of different datasets between the two models, only chicken breast and the optimized validation groups are shown in the multivariable analysis presented in Fig. 6. Here, PC1 and PC2 collectively explained approximately 75.8 % (65.5 % for PC1 and 10.3 % for PC2; full scatter matrix shown in Fig. S3B) of the variability. Although chicken breast samples formed a distinct cluster along PC1 (Fig. 6A), optimized samples from groups w/tensile BO and w/o tensile BO8 clustered closer to chicken breast. In contrast, the fully iterated BO without tensile data (w/o tensile BO12) and both RSM-optimized samples clustered further away. This indicates that BO, when incorporating tensile strength data, efficiently achieved optimal predictive conditions within just 7 iterations, matching the accuracy of the maximum iterative process. These findings underscore the better efficiency and accuracy of BO compared to RSM for this case study. Compared to Fig. 5B, Fig. 6B reveals a clearer relationship between validation samples and chicken breast. All extrudate samples exhibit greater hardness and chewiness than chicken breast. In contrast to Fig. 5C, tensile properties, particularly in the longitudinal direction (Tensile_L), became less influential in Fig. 6C, whereas springiness, hardness, and chewiness emerged as the top three discriminative properties. This suggests that tensile properties play a crucial role during initial optimization stages, while compression-related properties, such as springiness and hardness, become more significant during subsequent fine-tuning. The associated heatmap (Fig. 6C) further indicates greater textural similarity between samples optimized with tensile data using BO (w/tensile BO) and chicken breast, evidenced by the prevalence of lower values (bluer hues) in springiness, hardness, and chewiness.

## 4 Conclusions

This study demonstrates that Bayesian optimization (BO) is a powerful approach for optimizing high moisture extrusion process using a formulation of soy protein concentrate and wheat gluten. While Response Surface Methodology (RSM) provided a structured experimental design, its reliance on polynomial regression limited its ability to handle complex and noisy datasets. In contrast, BO achieved higher prediction accuracy with fewer experimental trials, particularly when tensile strength was incorporated as an additional response variable. Among all measured properties, tensile strength emerged as the most influential driver during the initial stages of optimization. These findings highlight the value of expanding the conventional property space, which is typically limited to compression and cutting tests, to include tensile properties that better reflect structural integrity and we foresee further expansion to include other linear and nonlinear material properties.

Looking ahead, future studies should implement BO beyond predefined experimental datasets and with more flexibility in the number of input parameters, such as screw speed and feeding material speed. Doing so will allow the algorithm to fully explore the design space and would further enhance optimization performance. In summary, this machine learning-based optimization method holds great potential for broader real-world applications in other complex food processing systems. However, the performance of BO, like other machine learning algorithms, can be influenced by factors such as program initialization and experimental noise, which should be carefully considered in future studies.

## CRediT authorship contribution statement

**Yingfen Jiang:** Conceptualization, Methodology, Investigation, Formal analysis, Data curation, Writing – original draft. **Noor Irsyad Bin Noor Azlee:** Investigation, Formal analysis, Data curation, Writing – original draft. **Wing Shan Ko:** Investigation, Formal analysis. **Kaiqi Chen:** Conceptualization, Data curation, Software development. **Bee Gim Lim:** Methodology, Funding acquisition. **Arif Z. Nelson:** Conceptualization, Supervision, Writing – review & editing, Funding acquisition.

## Declaration of generative AI and AI-assisted technologies in the writing process

During the preparation of this work the authors used ChatGPT in order to improve readability. After using this tool/service, the authors reviewed and edited the content as needed and take full responsibility for the content of the publication.

## Declaration of competing interest

The authors declare that they have no known competing financial interests or personal relationships that could have appeared to influence the work reported in this paper.

## Data Availability

Data will be made available on request.
