# Supplementary material for: Plant-based protein extrusion optimization: Comparison between machine learning and conventional experimental design
Source: Curr Res Food Sci. 2025 Jul 28;11:101157. doi: 10.1016/j.crfs.2025.101157 (PMC12336687; doi:10.1016/j.crfs.2025.101157)
Supplement: Multimedia component 1 [file mmc1.docx]

**Supplementary**

**Plant-based protein extrusion optimization: Comparison between machine learning and conventional experimental design**

Yingfen Jiang^a^, Noor Irsyad Bin Noor Azlee^a^, Wing Shan Ko^a^, Kaiqi Chen^b^, Bee Gim Lim^a^, Arif Z. Nelson^a, *^

^a^ Food, Chemical and Biotechnology Cluster, Singapore Institute of Technology, 1 Punggol Coast Road, Singapore, 828608, Singapore

^b^ Department of Computer Science, National University of Singapore, 13 Computing Drive, Singapore, 117417, Singapore

*Corresponding author at: Food, Chemical and Biotechnology Cluster, Singapore Institute of Technology, 1 Punggol Coast Road, Singapore, 828608, Singapore.

E-mail address: arif.nelson@singaporetech.edu.sg


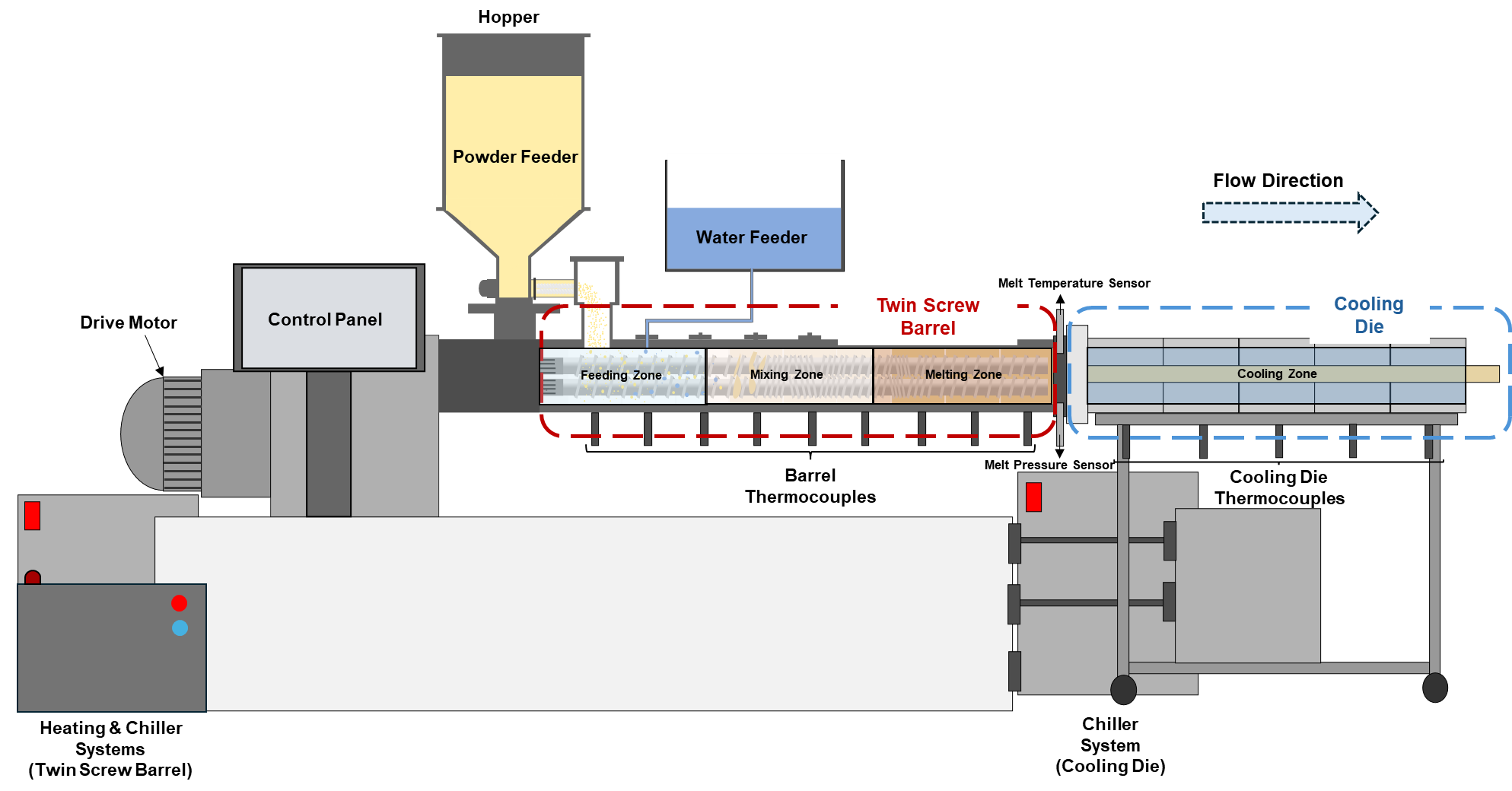


**Fig. S1**. Detailed schematic representation of a twin-screw extruder equipped with a cooling die.

**Table S1. Extruder system conditions during sample collection**

| Run | Barrel Temperature (°C) | Water Content (%) | Cooling Die Temperature (Section a, b) (°C) | Motor Torque (%) | Melt Temperature (°C) | Melt Pressure (bar) |
| --- | --- | --- | --- | --- | --- | --- |
| 1 | 110 | 55 | 70, 60 | 10 | 103 | 24 |
| 2 | 140 | 55 | 70, 60 | 9 | 129 | 19 |
| 3 | 110 | 65 | 70, 60 | 7 | 102 | 10 |
| 4 | 140 | 65 | 70, 60 | 7 | 126 | 9 |
| 5 | 110 | 60 | 60, 55 | 8 | 101 | 17 |
| 6 | 140 | 60 | 60, 55 | 8 | 127 | 13 |
| 7 | 110 | 60 | 80, 65 | 8 | 103 | 16 |
| 8 | 140 | 60 | 80, 65 | 7 | 128 | 8 |
| 9 | 125 | 55 | 60, 55 | 9 | 114 | 22 |
| 10 | 125 | 65 | 60, 55 | 6 | 113 | 9 |
| 11 | 125 | 55 | 80, 65 | 8 | 115 | 17 |
| 12 | 125 | 65 | 80, 65 | 7 | 115 | 9 |
| 13 | 125 | 60 | 70, 60 | 8 | 115 | 15 |
| 14 | 125 | 60 | 70, 60 | 8 | 113 | 15 |
| 15 | 125 | 60 | 70, 60 | 7 | 114 | 13 |


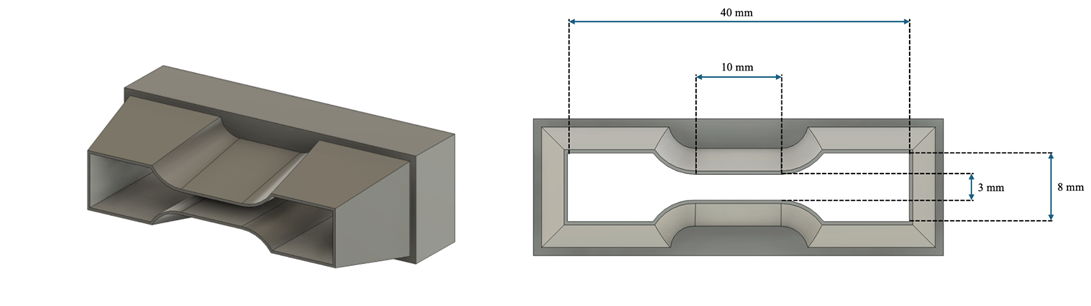


**Fig. S2.** Geometry of a dog-bone-shaped mold.

**Table S2.** Experimental values of mechanical properties

| **Run** | **B. Temp** | **Target WC** | **CD. Temp** | **Toughness, L (N)** | **Toughness, T (N)** | **Hardness (N)** | **Springiness** | **Chewiness (N)** | **Tensile Strength, L (N)** | **Tensile Strength, T (N)** |
| --- | --- | --- | --- | --- | --- | --- | --- | --- | --- | --- |
| 1 | 110 | 55 | 70 | 10.46 ± 0.77a | 11.12 ± 0.19a | 171.56 ± 10.86a | 0.86 ± 0.01a | 100.93 ± 10.59b | 2.82 ± 0.30b | 4.59 ± 0.34a |
| 2 | 140 | 55 | 70 | 10.43 ± 0.28a | 10.15 ± 0.31a | 179.15 ± 5.84a | 0.92 ± 0.03a | 133.59 ± 17.04a | 5.27 ± 0.47a | 4.82 ± 0.60a |
| 3 | 110 | 65 | 70 | 5.99 ± 0.11c | 6.32 ± 0.10d | 70.10 ± 3.62f | 0.84 ± 0.03a | 36.14 ± 2.83e | 0.95 ± 0.08d | 0.86 ± 0.03d |
| 4 | 140 | 65 | 70 | 5.49 ± 0.23d | 6.41 ± 0.35d | 106.21 ± 7.30d | 0.91 ± 0.04a | 67.44 ± 9.54c | 1.64 ± 0.19c | 1.31 ± 0.14d |
| 5 | 110 | 60 | 60 | 7.91 ± 0.37b | 8.33 ± 0.55c | 107.83 ± 7.25d | 0.86 ± 0.03a | 56.24 ± 7.80d | 1.51 ± 0.16c | 2.09 ± 0.05c |
| 6 | 140 | 60 | 60 | 8.31 ± 0.65b | 8.05 ± 0.05c | 150.41 ± 2.29b | 0.92 ± 0.02a | 103.94 ± 3.93b | 2.54 ± 0.09b | 2.08 ± 0.28c |
| 7 | 110 | 60 | 80 | 7.40 ± 0.45b | 9.48 ± 0.24b | 123.04 ± 4.19c | 0.92 ± 0.07a | 75.37 ± 8.12c | 1.04 ± 0.05d | 1.76 ± 0.23c |
| 8 | 140 | 60 | 80 | 7.30 ± 0.22b | 7.48 ± 0.08c | 113.39 ± 3.12d | 0.92 ± 0.02a | 77.46 ± 4.98c | 1.88 ± 0.06c | 1.87 ± 0.34c |
| 9 | 125 | 55 | 60 | 10.24 ± 0.81a | 10.68 ± 0.48a | 157.63 ± 5.57b | 0.90 ± 0.00a | 105.91 ± 6.42b | 3.07 ± 0.13b | 3.18 ± 0.53b |
| 10 | 125 | 65 | 60 | 5.81 ± 0.19c | 6.64 ± 0.22d | 73.90 ± 0.80f | 0.88 ± 0.01a | 40.93 ± 1.31d | 0.84 ± 0.12d | 0.92 ± 0.11d |
| 11 | 125 | 55 | 80 | 7.81 ± 0.61b | 8.11 ± 0.54c | 124.15 ± 7.57c | 0.89 ± 0.02a | 76.71 ± 8.02c | 2.95 ± 0.07b | 3.91 ± 0.43b |
| 12 | 125 | 65 | 80 | 6.45 ± 0.26c | 7.01 ± 0.45d | 68.38 ± 1.64f | 0.84 ± 0.01a | 33.50 ± 0.97e | 0.90 ± 0.14d | 1.22 ± 0.15d |
| 13 | 125 | 60 | 70 | 8.27 ± 0.41b | 8.01 ± 0.16c | 141.65 ± 5.31b | 0.88 ± 0.02a | 87.30 ± 1.22b | 1.88 ± 0.30c | 2.15 ± 0.32c |
| 14 | 125 | 60 | 70 | 8.10 ± 0.23b | 9.63 ± 0.62b | 137.08 ± 5.29c | 0.93 ± 0.06a | 80.78 ± 3.67c | 1.67 ± 0.20c | 2.29 ± 0.20c |
| 15 | 125 | 60 | 70 | 6.38 ± 0.11c | 7.19 ± 0.22d | 103.34 ± 4.99d | 0.91 ± 0.01a | 63.74 ± 4.38c | 1.27 ± 0.13d | 1.35 ± 0.23d |
| w/o_BO8 | 113 | 65 | 80 | 6.78 ± 0.43c | 7.76 ± 0.03d | 135.14 ± 4.38e | 0.85 ± 0.01a | 65.71 ± 5.40d | 1.37 ± 0.08e | 2.20 ± 0.15d |
| w/o_BO12 | 121 | 65 | 80 | 6.36 ± 0.29c | 6.91 ± 0.35d | 79.23 ± 5.44e | 0.84 ± 0.06a | 36.81 ± 3.67d | 0.89 ± 0.03d | 1.22 ± 0.05d |
| w/o_RSM | 117 | 65 | 80 | 6.44 ± 0.30d | 6.46 ± 0.20d | 94.99 ± 0.49e | 0.83 ± 0.04a | 41.08 ± 3.22d | 1.12 ± 0.06d | 1.17 ± 0.15d |
| w/_BO | 110 | 65 | 80 | 6.01 ± 0.35d | 6.83 ± 0.26d | 90.22 ± 4.68f | 0.88 ± 0.04b | 42.95 ± 1.85e | 0.73 ± 0.06d | 0.86 ± 0.09d |
| w/_RSM | 115 | 65 | 80 | 6.18 ± 0.09c | 7.15 ± 0.18d | 93.66 ± 5.16e | 0.85 ± 0.03b | 49.60 ± 2.97d | 1.23 ± 0.08d | 1.00 ± 0.08d |
| 0 | Chicken Breast | | | 4.61 ± 0.43d | 7.36 ± 0.68c | 25.60 ± 0.79g | 0.73 ± 0.04b | 9.41 ± 0.50f | 0.11 ± 0.02f | 0.62 ± 0.05d |

Note: RSM: response surface methodology. BO: Bayesian optimization. BO8/12: The 8^th^/12^th^ iteration from Bayesian optimization. w/o: without tensile properties; w/: with tensile properties. Different letters within the same column indicate statistically significant differences (*p* < 0.05)

**Table S3. Optimal extrusion conditions for model validation**

| Run | Barrel Temperature (°C) | Water Content (%) | Cooling Die Temperature (Section a, b) (°C) | Motor Torque (%) | Melt Temperature (°C) | Melt Pressure (bar) |
| --- | --- | --- | --- | --- | --- | --- |
| Modeling without tensile strength | | | | | | |
| RSM | 117 | 65 | 80, 65 | 7 | 108 | 11 |
| BO8 | 113 | 65 | 80, 65 | 7 | 104 | 10 |
| BO12 | 121 | 65 | 80, 65 | 7 | 111 | 11 |
|  |  |  |  |  |  |  |
| Modeling with tensile strength | | | | | | |
| RSM | 115 | 65 | 80, 65 | 7 | 106 | 10 |
| BO7 | 110 | 65 | 80, 65 | 7 | 102 | 9 |
| BO12 | 110 | 65 | 80, 65 | 7 | 102 | 9 |

Note: RSM: response surface methodology; BO7/8/12: The 7^th^/8^th^/12^th^ iteration from Bayesian optimization.


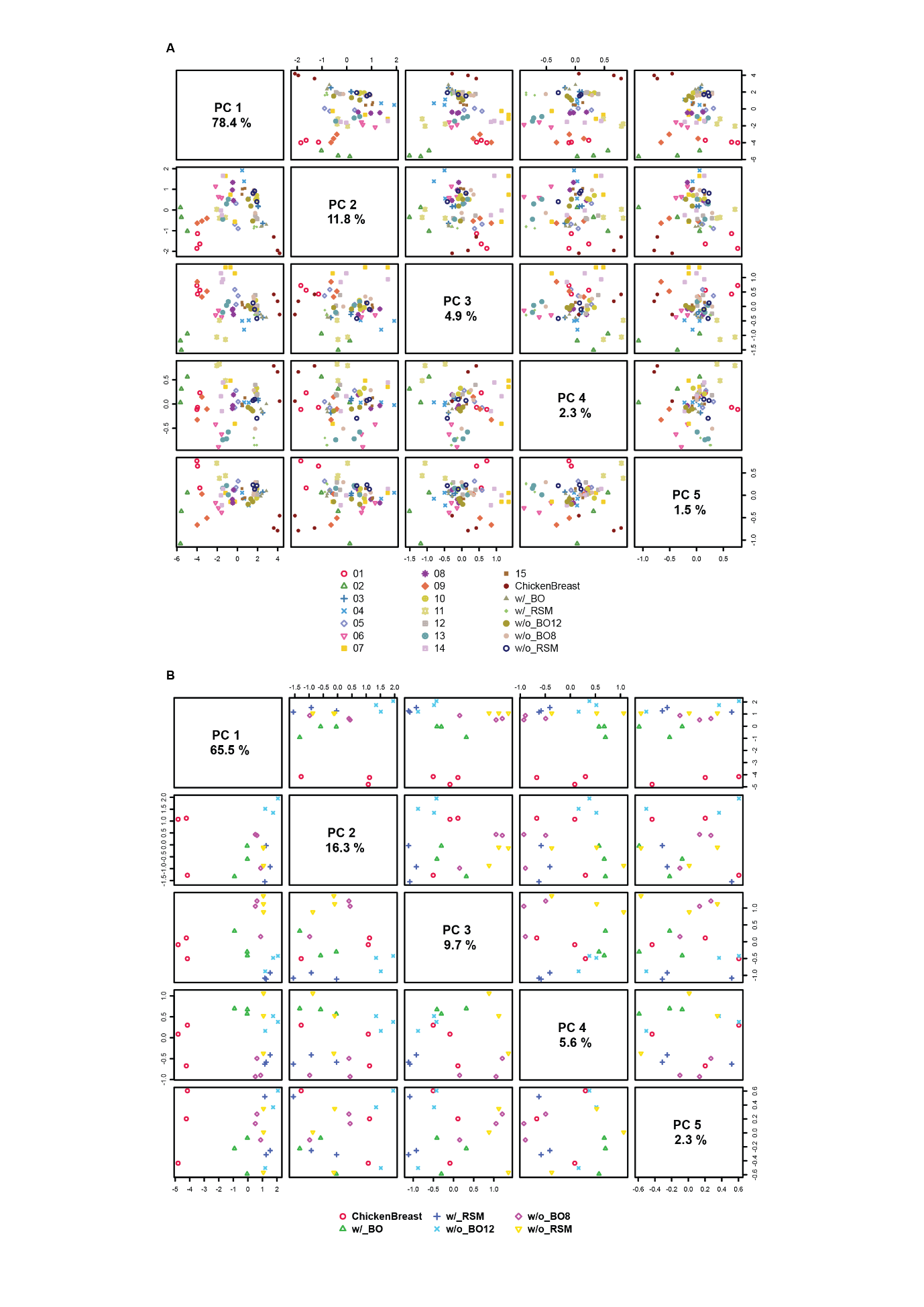


**Fig. S3.** The first five components for Principal Component Analysis across all groups (A) and optimized groups (B).
